# Telestration with augmented reality for visual presentation of intraoperative target structures in minimally invasive surgery: a randomized controlled study

**DOI:** 10.1007/s00464-022-09158-1

**Published:** 2022-03-09

**Authors:** C. Wild, F. Lang, A. S. Gerhäuser, M. W. Schmidt, K. F. Kowalewski, J. Petersen, H. G. Kenngott, B. P. Müller-Stich, F. Nickel

**Affiliations:** 1 Department of General, Visceral, and Transplantation Surgery, Heidelberg University Hospital, Im Neuenheimer Feld 420, 69120 Heidelberg, Germany; 2 Department of Urology, University Medical Center Mannheim, Heidelberg University, Theodor-Kutzer-Ufer 1-3, 68167 Mannheim, Germany; 3 German Cancer Research Center, 69120 Heidelberg, Germany

**Keywords:** Laparoscopy, Minimally invasive surgery, Augmented reality, Surgical training, Telestration, Cholecystectomy

## Abstract

**Aims:**

In minimally invasive surgery (MIS), intraoperative guidance has been limited to verbal communication without direct visual guidance. Communication issues and mistaken instructions in training procedures can hinder correct identification of anatomical structures on the MIS screen. The iSurgeon system was developed to provide visual guidance in the operating room by telestration with augmented reality (AR).

**Methods:**

Laparoscopic novices (*n* = 60) were randomized in two groups in a cross-over design: group 1 trained only with verbal guidance first and then with additional telestration with AR on the operative screen and vice versa for group 2. Training consisted of laparoscopic basic training and subsequently a specifically designed training course, including a porcine laparoscopic cholecystectomy (LC). Outcome included time needed for training, performance with Global Operative Assessment of Laparoscopic Skills (GOALS), and Objective Structured Assessment of Technical Skills (OSATS) score for LC, complications, and subjective workload (NASA-TLX questionnaire).

**Results:**

Telestration with AR led to significantly faster total training time (1163 ± 275 vs. 1658 ± 375 s, *p* < 0.001) and reduced error rates. LC on a porcine liver was performed significantly better (GOALS 21 ± 5 vs. 18 ± 4, *p* < 0.007 and OSATS 67 ± 11 vs. 61 ± 8, *p* < 0.015) and with less complications (13.3% vs. 40%, *p* < 0.020) with AR. Subjective workload and stress were significantly reduced during training with AR (33.6 ± 12.0 vs. 30.6 ± 12.9, *p* < 0.022).

**Conclusion:**

Telestration with AR improves training success and safety in MIS. The next step will be the clinical application of telestration with AR and the development of a mobile version for remote guidance.

**Supplementary Information:**

The online version contains supplementary material available at 10.1007/s00464-022-09158-1.

## Background

Minimally invasive surgery (MIS) has become the standard of care for many operations in abdominal and thoracic surgery. In recent times, even more advanced procedures such as pancreaticoduodenectomy are frequently performed laparoscopically [1]. MIS offers several advantages for the patient, including reduced tissue trauma and blood loss, shorter hospital stay, earlier return to work, and improved short-term quality of life [1]. However, MIS is more complex to learn for surgeons and the learning curve is longer, hence special laparoscopic training is needed [2, 3]. Basic skills can be well trained outside the operating room (OR) [2–4]. The necessary intraoperative training in MIS is still accompanied by certain risks and potential complications [5]. MIS comes with clear advantages for patients, but results in additional difficulties for surgeons especially during the learning phase that must be considered. In laparoscopic procedures, surgeons do not see the surgical field directly and cannot palpate the organs with their hands. Interaction between surgeons at the operating table is limited to verbal communication. In contrast to open surgery, in MIS the experienced surgeons cannot guide trainees with their hands in the operative field. In addition, previous experience in open surgery can only be transferred to MIS with limitations [6–8]. Unlike for open surgeries, during MIS the instructing surgeon cannot show the target structures directly in situ, instead having to verbally describe them. This can lead to problems in communication in the OR with the potential to prolong operative times, to cause stress, to worsen training, and to endanger patient safety and the success of the operation. In order to establish unequivocal communication, visual guidance would be needed on the MIS screen to provide optimal training support. Within this study, a new training concept for minimally invasive surgery was tested, for which trainees are supported with the help of telestration with augmented reality (AR): the iSurgeon system. This device allows the combination of verbal instructions during training with visual instructions on the laparoscopic monitor. The experienced surgeons can guide the trainees with their virtual hand overlayed on the MIS screen and can thus visually guide the trainees through the operation (Supplementary video 1).

The aim of the present study was to assess potential of telestration with AR in a preclinical yet realistic MIS training setting, including MIS basic skills, operative skills, cadaveric laparoscopic cholecystectomy (LC), and subjective workload.

## Materials and methods

### Setting and participants

This study was conducted within a voluntary elective course for medical students in their clinical years (3^rd^ to 6^th^ year of study) at Heidelberg University Medical School, Germany. Training took place in the MIS training center of the Department for General, Visceral, and Transplantation Surgery. Exclusion criteria were prior participation in laparoscopic courses as well as prior minimally invasive training time exceeding two hours. Sample size was determined according to the preliminary results of a pilot study. The pre-defined difference was calculated from the difference in the endpoint (time) from this pilot study. Sample size was determined under the assumption of *a* = 0.05 and power of 1 − *b* = 0.90, which resulted in a group size of 27 people (*µ*_1_ = 214, *µ*_2_ = 183, SD = 35) [9]. In order to compensate for possible drop-outs, group size was determined with 30 participants. After signing the consent form, participants were randomized into two groups. Randomization (block size 4) was performed by an independent employee, otherwise not involved in planning, conducting, or analyzing the study, using Research randomizer (http://www.randomizer.org). Randomization results were kept in sealed, opaque, and sequentially numbered envelopes until students were allocated by the main coordinator. Group 1 completed each task first with only verbal instructions, receiving additional help with AR only in their second round. Group 2 completed their first round with the help of AR, receiving only verbal instructions in their second round, respectively, in accordance with the crossover design. The study was approved by the local Ethics Committee at Heidelberg University (Code S-436/2018).

### Materials

**Fig. 1:**
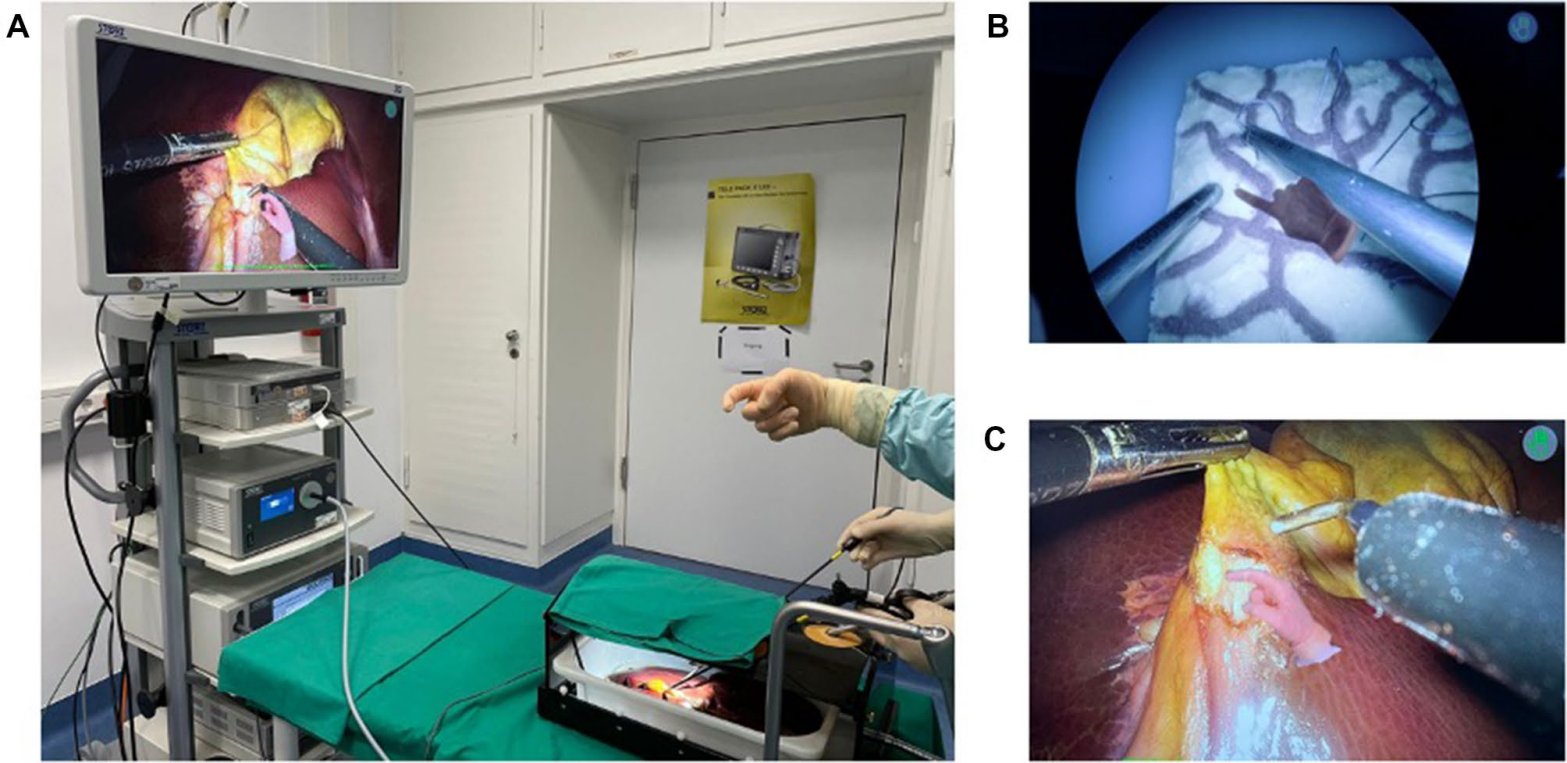
Visual guidance in the operating room by telestration with AR with the iSurgeon system. **A** Experimental setup of laparoscopic cholecystectomy on a porcine liver. The liver is placed in a plastic box within the box trainer. **B** The virtual hand of the experienced surgeon can be captured in the sterile field and displayed in real time on the operating screen. **C** Application of the iSurgeon system in a laparoscopic cholecystectomy in the box trainer. The transparency of the hand can be adapted (here 80%)

This study was performed on a Szabo–Berci–Sackier Box Trainer and a standard laparoscopy tower (KARL STORZ GmbH & Co. KG, Tuttlingen, Germany). The individual task stations were specifically constructed for this study. All used silicone models were specifically constructed for this study with EcoflexTM 00–30 (Smooth-On, Inc., Pennsylvania, USA) in the FabLab of Surgery at the University Hospital Heidelberg, Germany. The iSurgeon telestration system of AR-based video assistance was developed at the Department of General, Visceral, and Transplantation Surgery at Heidelberg University Hospital within the realm of a federally funded EXIST program and was provided for this study (Fig. 1). For LC a fenestrated grasper, curved scissors, clip applicators, and laparoscopic monopolar hook electrode were used (KARL STORZ GmbH & Co. KG, Tuttlingen, Germany).

### Study design and setting

**Fig. 2:**
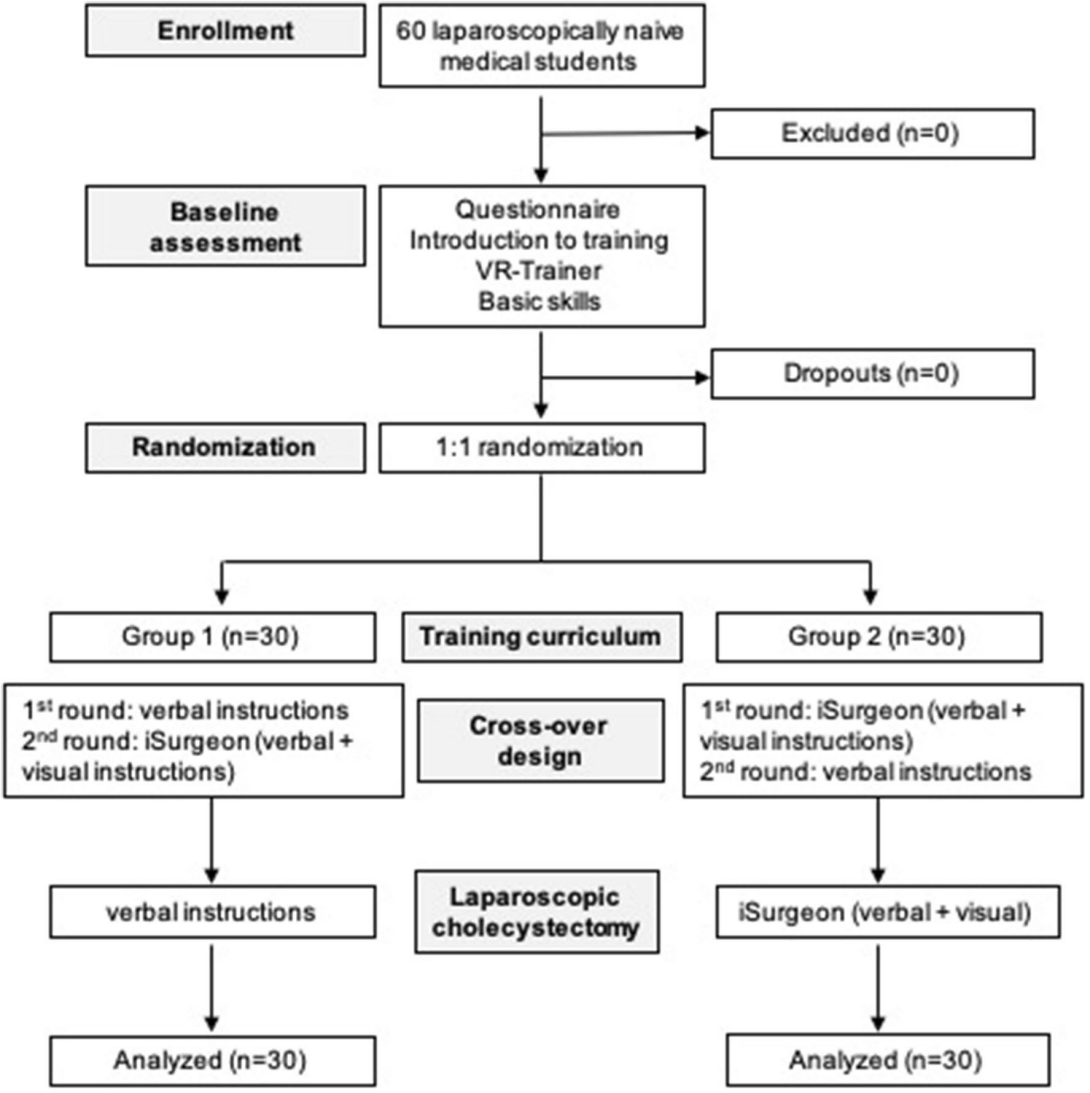
Flowchart illustrating the process of the study reported in line with the CONSORT criteria

This study is a randomized controlled, monocentric study with crossover design (Fig. 2). Prior to training, all 60 participants were asked to complete the Mental Rotation Test A (MRT-A), which analyzes spatial thinking [10]. Within a pretest, participants practiced with 6 tasks from the basic module (Task 3–8) on a Virtual Reality (VR) Trainer (LAP Mentor III, 3D Systems, Rock Hill, USA). Data collected on the VR Trainer were evaluated with the Heidelberg VR Score [4]. Subsequently, trainees completed basic laparoscopic training on the box trainer, consisting of two PEG transfers and threading rubber bands through multiple eyelets.

The actual training course consisted of 9 stations/tasks: 1- PEG transfer, 2- Marking boxes, 3- Abstract figure, 4- Nail course, 5- Silicone reins, 6- Intestine loops, 7- Blood vessels, 8- Tissue exposition, and 9- Resection lines (2 rounds per group as per crossover design, one with verbal instructions, one with use of telestration with AR). Lastly, participants completed a LC on a porcine liver. Additional visual support with AR was provided by an instructor, whose virtual hands were projected via AR onto the laparoscopic screen in real time (Fig. 1). The whole study was performed by the same tutor, who was specially trained in MIS and LC.

#### Laparoscopic cholecystectomy on a porcine liver

In order to demonstrate the ability to transfer learned skill into an intraoperative setting, participants completed a LC on a porcine liver in the box trainer at the end of the trial [11]. Group 1 completed the operation with only verbal instructions, while group 2 had additional visual support from the AR system. The procedure split into two parts: firstly, the cystic duct and cystic arteria had to be identified, exposed, clipped, and severed (maximum 45 min). Secondly, the gallbladder was removed from the gall bladder bed (maximum 45 min) (Fig. 1A).

### Outcome parameters

The primary endpoint of this study was defined as total training time for the training course. Moreover, error rates were analyzed. Error events were defined based on a series of pilot trials and subsequent expert discussion. An additional goal of this study was to determine if the use of AR led to a change in subjective workload. Therefore, participants were asked to fill out the National Aeronautics and Space Administration-Task Load Index (NASA-TLX) questionnaire after each round of tasks 4–9. The NASA-TLX is a validated test to measure workload and stress [12].

For LC respective times were measured and the instructor evaluated the procedure in real time according to the following scores: Global Operative Assessment of Laparoscopic Skills (GOALS) Score, task-specific GOALS Score, and Objective Structured Assessment of Technical Skills (OSATS) Score (global and task specific).These scores are validated check lists according to which several areas of surgical performance can be evaluated [13, 14]. Additional points of evaluation were damage to liver tissue, perforation of gall bladder or damage to blood vessels, as well as correct placement of clips. In order to compare procedures, given anatomical differences between livers, the size of the gall bladder bed was measured and the difficulty of the procedure was rated on a scale of 1–10. The criteria for the rating included fatty tissue surrounding the blood vessels (little vs. a lot of fat), condition of the connective tissue and peritoneum (delicate vs. crude), position of the gall bladder within the liver (superficial vs. deep), as well as anatomical aberrations.

### Statistical analysis

Statistical analysis was performed in collaboration with the Department of Medical Biometry and Informatics at Heidelberg University, Germany. All data were entered into a spreadsheet and statistical analysis was performed using SPSS (version 25.0, IBM SPSS Inc., Chicago, Illinois, USA). Since the study was conducted in a crossover design, participants represented their own control group (training without/with AR) and differences in basic traits between the groups did not have any relevant effect on the results. Differences between the two groups were calculated with the help of the *t* test for independent random sampling and examined with the help of the Chi-squared test. A *p*-value of ≤ 0.05 was considered statistically significant.

## Results

All participants successfully completed the entire trial and no participant was excluded. After randomization, both groups showed similar demographic distribution. Regarding pretest data collected in the beginning of the study with MRT-A questionnaires, no difference was seen between the two groups (Supplementary Table 1).

### Primary endpoint

**Fig. 3:**
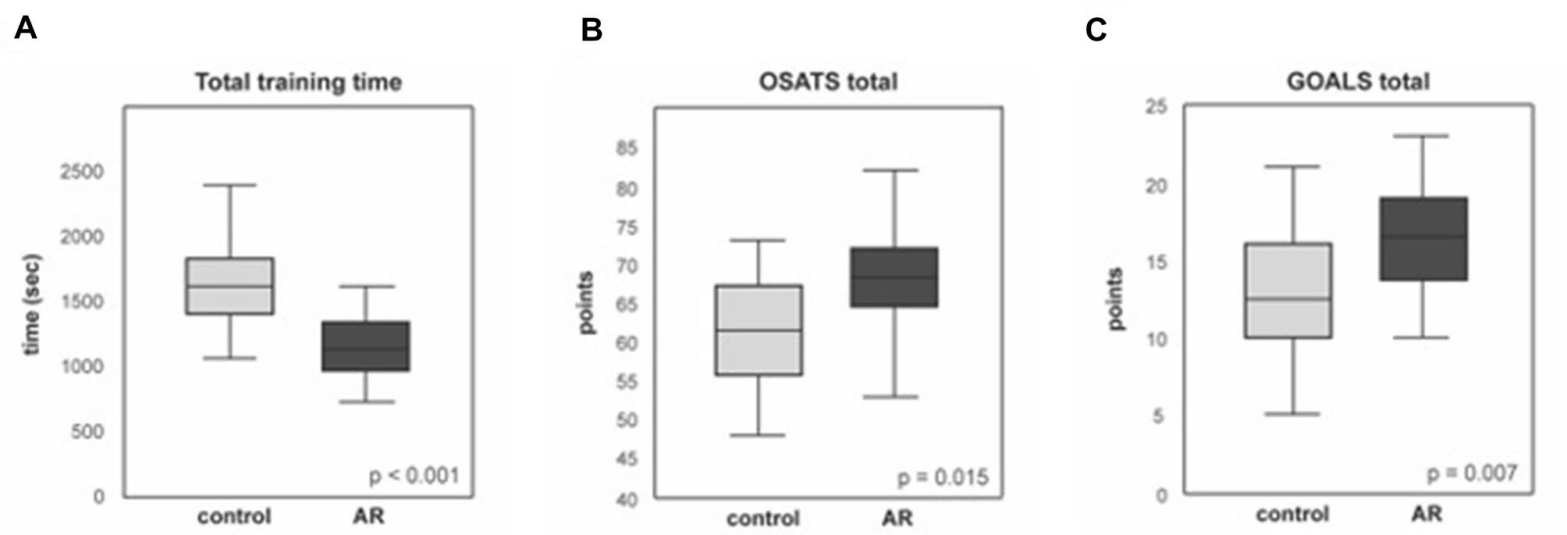
**A** Total training time without (control) and with AR for training course. **B** Total OSATS score without (control) and with AR for laparoscopic cholecystectomy. **C** Total GOALS without (control) and with AR for laparoscopic cholecystectomy. *Significant for *p* < 0.05, *t*-test

Telestration with AR led to significantly faster completion of all tasks and to reduction of overall training time needed to complete the tasks of − 29.8% (Fig. 3A).

### Secondary endpoints

AR was able to significantly reduce the error rate in most tasks. In tasks where points were distributed, AR raised the number of scored points (Table 1). These data clearly demonstrate that usage of AR during laparoscopic training leads to an altogether better performance of participants when compared to verbal guidance only. In particular, when drawing abstract figures participants scored significantly more points when instructed with AR compared to sole verbal instructions. Size, angles, as well as size relation of the figures made under guidance of telestration with AR were superior to outcome with only verbal instructions. Interestingly, this was true for both groups. This means, even if the first round was performed with AR and participants had a chance to get a feeling for correct angels and relations, they scored less points in the second round with solely verbal guidance. The differences in verbal and AR-assisted instructions are clearly visible (Supplementary Figure 1).

**Table 1:** Time and error rate/total scored points in tasks (s) without AR (control) and with AR (mean value ± SD), * significant for *p* < 0.05, *t* test

	Control (mean ± SD)	With AR (mean ± SD)	Difference	*p*-value
*Time*				
PEG Transfer	233 ± 69	203 ± 61	− 12.8%	< 0.001*
Marking boxes	110 ± 23	68 ± 18	− 38.2%	< 0.001*
Abstract figure	116 ± 31	75 ± 22	− 35.3%	< 0.001*
Nail course	164 ± 40	101 ± 19	− 38.4%	< 0.001*
Silicone reins	171 ± 33	117 ± 26	− 31.6%	< 0.001*
Intestine loops	305 ± 143	242 ± 113	− 20.7%	< 0.001*
Blood vessels	128 ± 24	61 ± 16	− 52.3%	< 0.001*
Tissue exposition	226 ± 60	164 ± 46	− 27.4%	< 0.001*
Resections lines	205 ± 74	132 ± 49	− 35.6%	< 0.001*
*Error rate / scored points*				
PEG transfer (errors)	1.5 ± 1.6	0.0 ± 0.0		< 0.001*
Marking boxes (errors)	0.5 ± 0.9	0.0 ± 0.0		< 0.001*
Abstract figure (points)	14.3 ± 2.6	18.6 ± 0.7		< 0.001*
Nail course (errors)	3.3 ± 2.0	0.2 ± 0.5		< 0.001*
Silicone reins (errors)	0.1 ± 0,6	0.1 ± 0.3		0.203
Blood vessels (errors)	1.2 ± 0.9	0.0 ± 0.0		< 0.001*
Tissue exposition (errors)	0.4 ± 0.9	0.0 ± 0.0		0.001*
Resection lines (points)	12.9 ± 2.4	14.2 ± 2.4		< 0.001*

### Laparoscopic cholecystectomy

For LC on a porcine liver, the level of difficulty, and the size of gall bladder bed, no differences between the two groups were determined (difficulty 5 ± 2 vs. 4 ± 2, *p* = 0.336 and size of gallbladder bed in cm^2^ 21 ± 4 vs. 22 ± 3, *p* = 0.455). Accordingly, both groups completed the surgery in comparable time (80 ± 14 vs. 77 ± 14, *p* = 0.458).

The GOALS and OSATS scores (Supplementary Table 2 and Fig. 3B, C) showed significantly better surgical performance with telestration with AR than without. There were less complications (damage on liver tissue or vessels or misplaced clips) with AR than without AR (60% vs. 86.7%, *p* = 0.020) (Supplementary Table 3).

### Subjective self-evaluation

Results of NASA TLX questionnaire show that participants experienced training with the help of AR to be less challenging, mentally as well as physically. Moreover, participants felt significantly more relaxed and rated themselves as more successful when AR was used. Additionally, participants felt less time pressure when training with AR (Table 2).

**Table 2:** Results of NASA TLX questionnaires without AR (control) and AR for skill tasks (mean value ± SD), * significant for *p* < 0.05, *t* test

	Control (mean ± SD)	with AR (mean ± SD)	*p*-value
How mentally demanding was the task?	48.9 ± 14.3	33.3 ± 14.8	< 0.001*
How physically demanding was the task?	38.1 ± 13.3	35.1 ± 13.8	0.002*
How much time pressure did you feel?	33.6 ± 12.5	30.6 ± 12.9	0.022*
How successful were you in accomplishing the task?	65.5 ± 10.4	71.5 ± 11.4	< 0.001*
How hard did you have to work to accomplish your level of performance?	67.6 ± 9.4	71.6 ± 9.6	< 0.001*

## Discussion

In the present study the use of telestration with AR led to a faster completion of tasks, better surgical performance, and less complications in a preclinical setting. This applied to the specifically designed laparoscopic training course as well as LC on a porcine liver, which was done with less complications and better GOALS and OSATS performance scores. Lastly, the data suggest that telestration with AR increases the subjective feeling of success and self-confidence of participants, while simultaneously reducing subjective workload.

Telestration with AR resulted in faster completion of training tasks and a significant reduction of training time. Time is a frequently used parameter to objectively measure learning curves and quality of a surgical procedure. However, the use of operative time as a performance parameter is also seen controversially. It is well known that long operation times generate higher costs for the hospital and can have a negative effect on patient’s outcome. For example, overly long operation times lead to an increased risk of post-operative wound infections, pneumonia, cardiopulmonary failure, or deep vein thromboses [15–17]. Moreover, prolonged operative time can cause exhaustion of the surgical team, thus further increasing the risk for complications. A study by Procter et al. determined an increased complication risk for each half hour of operation time in LC [18]. Cheng et al. consider decreased operative times as the main goal for surgeons, since their systematic review highlights a positive association between prolonged operative time and complications [16]. Then again the quality of a surgical outcome cannot be measured solely by operation time but rather depends on the applied operation technique, the individual difficulty of the case, and the surgeon’s experience. Longer operative time can also be a consequence of higher attentions to details and a more careful approach, which in turn leads to reduced complication rates. In the present study, real-time telestration with AR improved correct identification of target structures on the laparoscopic screen by trainees, thus significantly reducing training time. This was mainly due to simplified implementation of the tasks, as AR enabled the instructor to directly point out target structures on the laparoscopic screen, whereas solely verbal instructions have to be processed by the learning surgeon and can lead to misunderstandings and delayed recognition of target structures [19]. These findings are in line with other studies that show that remote surgical support and telementoring facilitate interaction between surgeons and particularly support the surgeon on site with topographic and anatomical issues [20–22]. Consequently, operating with the iSurgeon resulted in a significant reduction of total training time, as participants were able to identify target structures faster and more precisely. This indicates that the intraoperative application of the telestration AR system iSurgeon could help to reduce overall operation time, thereby contributing to patient safety during minimally invasive procedures.

In addition to operating time, the quality of a surgical procedure is largely determined by the surgeon’s ability to clearly identify anatomical target structures and work bimanually with laparoscopic tools as well as a general understanding of the surgical procedure. The experience and talent of the surgeon, as well as individual willingness to take risks also play key roles regarding a successful outcome [23]. In order to lower the rate of complications, communication inside the operating room has to be optimal, thereby improving guidance also for less-experienced surgeons. Studies show that problems in communication and misunderstandings between instructing surgeon and trainee can lead to intraoperative mistakes [24, 25]. In the present study, participants with AR-mediated visual guidance by telestration completed the laparoscopic tasks more precisely and made fewer mistakes compared to verbal instruction only. This applied especially to tasks with difficult anatomical proportions, for example, guiding a needle through defined areas in a net of silicone blood vessels. Strikingly, with application of AR not a single blood vessel was wrongly identified due to the excellent possibility for the tutor to guide the trainee, highlighting the advantage of AR-augmented guidance during training. With patient safety in mind, introducing telestration with AR into the operating room could be especially useful for training procedures, in order to prevent errors and possible complications and to increase the quality of patient care.

To test the transferability of these results to a more complex intraoperative setting, participants performed a LC on a porcine liver. LC is a commonly performed operation by junior surgeons. Introductory training of LC in box trainers and on a VR trainer has been shown to result in significant improvement regarding time and GOALS score when compared to no training [2]. Thus, LC in box trainers is considered as an effective tool to measure improvement of operative skills in trainees. Interestingly, the outcome in the present study was consistent with the previous findings from the training course: AR-supported guidance resulted in an improved and more precise performance with less complications. Surgical performance was scored with validated assessment tools for surgical skills with the OSATS and GOALS scores [2, 3, 26]. Strikingly, the application of the AR telestration system resulted in significantly higher scores in both total GOALS and total OSATS during LC. Interestingly, participants that were trained with AR scored higher in OSATS specific, but not in OSATS global. OSATS global assesses overall aspects of the LC, such as work flow, completion and tissue handling, while OSATS-specific rates operation steps that are closer associated with the identification of anatomical structures, such as the cystic duct, artery, or the Calot triangle. In particular, the iSurgeon telestration AR system facilitates identification of anatomical risk and target structures, which clearly reflect in a better OSATS-specific score. Participants performed the LC for the first time, thereby aspects of OSATS global such as tissue handling and work flow might have been challenging factors that could not be compensated with the application of AR. However, with AR-mediated guidance, participants performed significantly better in global, task specific, and total GOALS, thus suggesting an obvious benefit for the iSurgeon. Previous work suggests a benefit for multimodality in laparoscopic training, as training approaches that combine training modalities, e.g., box trainer and VR, improve the learning efficiency [2, 27]. Junior residents benefit more from multimodal training than experts, since the learning curve in laparoscopic surgery is particularly high in the beginning [2]. Thus, the application of AR in laparoscopic training may provide an advantage for inexperienced surgeons in particular but also for experts during complex and difficult procedures. Taken together, the results indicate that the better performance with AR during the training course can be translated to a more complex operative setting. Noticeably, operation time during LC was not improved by AR. One possible explanation for this observation could be that all participants were surgical novices with little previous operating skills and the operation on defrosted porcine liver is a relatively challenging surgical task regarding tissue handling as well as management of laparoscopic tools for the novice trainees. Overall, the AR telestration system improved training performance in the present study and thus presents a powerful tool to facilitate communication and guidance for young surgeons in the operating room.

Yurko et al. were able to show that subjective success and self-confidence play key roles in the subjective workload and that high subjective workload during laparoscopic procedures causes weaker performance, even from experienced surgeons [28]. The results from the present study suggest a significant reduction of intraoperative stress for trainees by usage of AR in the operative field. Participants who were guided with AR during training experienced significantly reduced mental and physical burden and performed significantly better when compared to solely verbal instructions. These results fit previous findings which state that a higher workload leads to quicker exhaustion and, subsequently, a decrease in the surgeon’s ability to focus. As a result, the number of mistakes as well as operation time can increase [29]. Hence, use of telestration with AR may improve efficiency and outcome quality of laparoscopic performances by also reducing workload and stress.

Training in MIS is currently performed outside the operating room on box trainers, VR trainers, animal models, and e-learning [3]. These training modalities are combined in multimodality training to their advantages [2]. Over the last years, the field of augmented reality in medicine has gained increasing importance, especially in MIS and robotic surgery [30]. Telestration with AR improves surgical training in the training setting and in the operating room. Telestration with AR also facilitates global communication in videoconferences and telesurgical assistance. The AIS Telesurgeon is a telestration system with AR that uses 5G to ensure a stable access to telesurgical assistance. This device allows communication between a mentor and the operating team on distant locations in real time [20]. A recent study examined the application of semitransparent ghost tools overlaid on the surgeon’s field of view in robotic surgery training. Results suggest this 3-dimensional proctoring device can serve as an effective mentoring tool [31]. Besides the iSurgeon, another technology that addresses the subject of telestration in MIS is Proximie. Proximie is a cloud-based AR telestration platform that can virtually connect surgeons in remote locations, thereby mimicking the situations in the OR [32]. However, to our knowledge, the current study is the first that shows a clear benefit for surgical training with AR-based telestration regarding operation time and error rate in a highly standardized setting in a randomized study.

## Limitations

The trainees were medical students in their clinical years with limited operating room experience. However, since the course was voluntary, only students with greater interest in surgery enrolled. In addition the medical students represent a homogenous study population and the study could be executed under very standardized training conditions. The study was performed on box trainers in a bright environment, a situation that is not entirely comparable with the largely dimmed light and sterile conditions in the operating room. Due to the nature of the study, blinding to group allocation was not possible and participants were directly observed. As a consequence, a possible selection bias has to be discussed. We believe that the cross-over design of our study is a powerful instrument to minimize the effects of such bias, as each subject acts as his or her own control. Generally, the AR-based iSurgeon telestration system can be easily integrated in the conventional operating room setting, as it is conceived for incorporation on the laparoscopic screen, thus maintaining the sterile environment. Naturally, the implementation of a new device is associated with costs for acquisition and maintenance. However, reduced operation time, minimization of surgical complications, and improvement of medical education subsequently lead to long-time benefits for patient safety as well as hospital economics. Usually, laparoscopic procedures are performed in a surgical team consisting of surgeons, anesthetists, and nursing staff, thereby resulting in different communication conditions than during training in the present study. This is rather an additional argument for use of telestration since the sound levels and distractions of surrounding conditions in the operating room are usually of higher level than in the training center and visual information therefore seems even more suitable. Therefore, we consider that the results of the present study can be translated into the operating room.

## Conclusion

The ability to instruct young surgeons with precision and efficiency presents a great challenge of MIS. Through the use of telestration AR in the present study the trainees completed laparoscopic exercises faster and performed a laparoscopic cholecystectomy better and with fewer complications compared to verbal instructions only. The trainees felt more assured and made fewer mistakes, while the instructing surgeon could guide the trainee more easily and effectively with visual guidance in addition to verbal guidance. Future studies will evaluate telestration with AR with the iSurgeon system in clinical practice as well as the mobile version for remote proctoring to improve patient care.

## Supplementary Information

Below is the link to the electronic supplementary material.

Supplementary file1 (JPG 65 KB)

Supplementary file2 (DOCX 14 KB)

Supplementary file3 (DOCX 14 KB)

Supplementary file4 (DOCX 15 KB)

Supplementary file5 (MP4 42378 KB)

## Abbreviations

AR: Augmented reality
GOALS: Global Operative Assessment of Laparoscopic Skills Score
LC: Laparoscopic cholecystectomy
MIS: Minimally invasive surgery
NASA TLX: NASA Task Load Index
OSATS: Objective Structured Assessment of Technical Skill
