# Supplementary figures and images for: Telestration with augmented reality for visual presentation of intraoperative target structures in minimally invasive surgery: a randomized controlled study
Source: Surg Endosc. 2022 Mar 9;36(10):7453–61. doi: 10.1007/s00464-022-09158-1 (PMC9485092; doi:10.1007/s00464-022-09158-1)

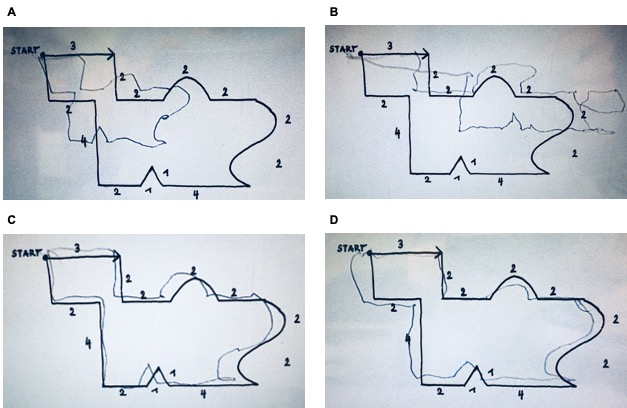

Supplement: Supplementary file 1 — Supplementary file1 (JPG 65 KB) [file 464_2022_9158_MOESM1_ESM.jpg]
