# Supplementary material for: Telestration with augmented reality for visual presentation of intraoperative target structures in minimally invasive surgery: a randomized controlled study
Source: Surg Endosc. 2022 Mar 9;36(10):7453–61. doi: 10.1007/s00464-022-09158-1 (PMC9485092; doi:10.1007/s00464-022-09158-1)
Supplement: Supplementary file 2 — Supplementary file2 (DOCX 14 KB) [file 464_2022_9158_MOESM2_ESM.docx]

|  | group 1 (*n* = 30) | | group 2 (*n* = 30) | |
| --- | --- | --- | --- | --- |
| sex (male) (mean value) | | 11 (36.7%) | | 18 (60%) |
| age (years) (mean value ± SD) | | 23 ± 3 | | 25 ± 4 |
| study year (mean value ± SD) | | 4 ± 1 | | 4 ± 1 |
| MRT-A (points) (mean value ± SD) | | 47 ± 1 | | 46 ± 2 |
| VR-Score (points) (mean value ± SD) | | 753 ± 112 | | 689 ± 140 |
| open surgeries seen (median) | | 10 | | 10 |
| laparoscopic surgeries seen (median) | | 2 | | 3 |

**Suppl. Table 1.** Baseline characteristics
