# Supplementary material for: Telestration with augmented reality for visual presentation of intraoperative target structures in minimally invasive surgery: a randomized controlled study
Source: Surg Endosc. 2022 Mar 9;36(10):7453–61. doi: 10.1007/s00464-022-09158-1 (PMC9485092; doi:10.1007/s00464-022-09158-1)
Supplement: Supplementary file 3 — Supplementary file3 (DOCX 14 KB) [file 464_2022_9158_MOESM3_ESM.docx]

|  | Control (mean ± SD) | with AR (mean ± SD) | p-value |
| --- | --- | --- | --- |
| GOALS Global | 14 ± 3 | 16 ± 3 | 0.011* |
| GOALS Task Specific | 4 ± 2 | 5 ± 2 | 0.010* |
| GOALS Total | 18 ± 4 | 21 ± 5 | 0.015* |
| OSATS Global | 22 ± 3 | 23 ± 4 | 0.148 |
| OSATS Specific | 39 ± 7 | 43 ± 7 | 0.011* |
| OSATS Total | 61 ± 8 | 67 ± 11 | 0.007* |

**Suppl. Table 2.** GOALS und OSATS scores without AR (control) and with AR, * significant for p<0.05, t-test
