# Supplementary material for: Telestration with augmented reality for visual presentation of intraoperative target structures in minimally invasive surgery: a randomized controlled study
Source: Surg Endosc. 2022 Mar 9;36(10):7453–61. doi: 10.1007/s00464-022-09158-1 (PMC9485092; doi:10.1007/s00464-022-09158-1)
Supplement: Supplementary file 4 — Supplementary file4 (DOCX 15 KB) [file 464_2022_9158_MOESM4_ESM.docx]

|  |  | Control (%) | | with AR (%) | | p-value |
| --- | --- | --- | --- | --- | --- | --- |
| Liver damage | no damage | | 16.7% | | 33.3% | 0.096 |
|  | minor damage | | 53.3% | | 56.7% |  |
|  | major damage | | 30.0% | | 10.0% |  |
| Gallbladder perforation | no damage | | 10.0% | | 26.7% | 0.214 |
|  | minor damage | | 30.0% | | 30.0% |  |
|  | major damage | | 60.0% | | 43.3% |  |
| Damage on artery/cystic duct | no damage | | 63.3% | | 70.0% | 0.825 |
|  | repairable damage | | 10.0% | | 10.0% |  |
|  | irreparable damage | | 26.7% | | 20.0% |  |
| Placement of clips | Correctly | | 26.7% | | 43.3% | 0.200 |
|  | Slightly out of place | | 53.3% | | 50.0% |  |
|  | Structure damaged, clips not closing | | 20.0% | | 6.7% |  |
| Combined end point | no damage on liver tissue, vessels or damage through placement of clips | | 13.3% | | 40% | 0.020* |
|  | major damage on liver tissue, vessels or damage through placement of clips | | 86.7% | | 60% |  |

**Suppl. Table 3:** Results of the laparoscopic cholecystectomy on a porcine liver w/o and with AR, * significant for p<0.05, t-test.
